# Sugar-Sweetened and Diet Beverage Consumption in Philadelphia One Year after the Beverage Tax

**DOI:** 10.3390/ijerph17041336

**Published:** 2020-02-19

**Authors:** Yichen Zhong, Amy H. Auchincloss, Brian K. Lee, Ryan M. McKenna, Brent A. Langellier

**Affiliations:** 1 Department of Epidemiology and Biostatistics, Dornsife School of Public Health, Drexel University, Philadelphia, PA 19104, USA; yz624@drexel.edu (Y.Z.); bkl29@drexel.edu (B.K.L.); 2 Department of Health Management and Policy, Dornsife School of Public Health, Drexel University, Philadelphia, PA 19104, USA; rmmckenna@gmail.com (R.M.M.); bal95@drexel.edu (B.A.L.)

**Keywords:** sugar-sweetened beverages, public policy, nutrition, epidemiology, public health

## Abstract

In January 2017, Philadelphia (Pennsylvania) implemented an excise tax ($ 0.015/ounce) on sugar-sweetened and diet beverages. This study is a general population-based study to report on the longer-term impacts of the tax on within-person changes in consumption 12 months after implementation. A quasi-experimental difference-in-difference design was used to contrast Philadelphia vs. nearby comparison cities (Trenton, New Jersey; Camden, New Jersey; and Wilmington, Delaware) at baseline (December 2016–January 2017) vs. 12-month follow-up (December 2017–February 2018). A random-digit-dialing phone survey was administered to a population-based cohort. Analyses assessed changes in 30-day consumption frequency and ounces of sugar-sweetened and diet beverages (and a substitution beverage, bottled water) in the analytic sample (N = 515). After 12 months, relative to the comparison group, Philadelphians were slightly more likely to decrease their frequency of sugar-sweetened beverage consumption (39.2% vs. 33.5%), and slightly less likely to increase their frequency of sugar-sweetened beverage consumption (38.9% vs. 43.0%). The effects of the tax estimated in the adjusted difference-in-difference analysis were very small (for example, changes in monthly sugar-sweetened beverage consumption in Philadelphia relative to comparison cities was −3.03 times or −51.65 ounces) and confidence intervals were very wide. Results suggested that, one year after implementation, there was no major overall impact of the tax on general population-level consumption of sugar-sweetened or diet beverages, or bottled water. Future studies should test whether the tax’s effect differs in vulnerable sub-populations.

## 1. Introduction

Sugar-sweetened beverages (SSB) are significant sources of added sugar and calories in the American diet [1,2]. High consumption of SSB is associated with increased risk of obesity, type 2 diabetes, cardiovascular disease and other health problems [3,4,5,6,7,8]. Reasons for high consumption include the low price of SSB relative to healthier beverages, high availability, and aggressive marketing [9,10,11]. A systematic literature review of food price elasticity found that the demand for SSB is responsive to price changes [12], which forms the basis for implementing a beverage tax to reduce consumption. 

Several municipalities (i.e., Berkeley, Boulder, SF, Seattle, Oakland, Albany) have enacted sugary beverage taxes as a way to generate needed revenue from sales of luxury/discretionary items [13], and as part of a multi-pronged approach to improve population health [14]. The Philadelphia beverage tax proposal was introduced with the explicit goal of financing universal pre-kindergarten programs and improvements to parks and recreation facilities, but did not mention potential benefits to health due to reduced SSB consumption [15]. The Philadelphia beverage tax was the largest beverage tax at the time it passed ($ 0.015/ounce) and has been in effect since 1 January 2017. Unlike other city-level beverage taxes that affect only SSB, the Philadelphia beverage tax covers both sugar-sweetened and diet beverages (SSDB). Recent reports have documented that, after one year, the Philadelphia beverage tax has been fully passed along to customers (97% pass-through rate) leading to an average of 34% price increase for taxed beverages [16]. 

Prior work describing the impacts of beverage taxes, have largely focused on changes in beverage purchases/sales, with most studies finding declines compared with predicted sales in the absence of the tax. One year following the tax, SSB sales declined by 9.6% in Berkeley, California, and by 22% in Seattle, Washington [17,18]. In Mexico, where cross-border purchases were minimal because the tax was implemented nationwide, a 7.6% decline was reported after two years of the tax [19]. Similar beverage tax in Catalonia reduced the prevalence of regular taxed beverage consumers by 39% [20]. A recent systematic review and meta-analysis of the real-world SSB tax impact found that a 10% price increase caused by the tax was associated with an average of 10% reduction in beverage purchases [21]. With more than a 30% price increase due to the Philadelphia beverage tax, two recent studies reported large declines in sales from 46% to 51% in Philadelphia, and from 22% to 24% after adjusting for cross-border shopping [16,22]. Another study reported that consumers decreased SSB purchase by 8.5 ounces per shopping trip in Philadelphia relative to its comparison communities [23]. However, a decline in sales or purchases within the taxed area does not necessarily translate into a reduction in consumption, which is a more public health-relevant outcome, because consumers may increase purchases of taxed beverages in nearby jurisdictions where beverages are not taxed (i.e., cross-border shopping). [16] In addition, the sales data do not capture out-of-home consumption and purchases from stores not participating in the large market research networks.

There is limited empirical evidence of beverage tax impacts on consumption in US contexts. Previously, our team examined the impact of the Philadelphia SSDB tax on consumption using a repeat cross-sectional design, and found a decrease in frequent sugary soda consumption and an increase in bottled water consumption shortly after tax implementation [24]. Similar short-term results were reported in another study conducted in Berkeley [25]. The results of longer-term impacts are mixed. Some studies have found declines in consumption [26,27] while other studies suggest that the impact on total SSB demand or consumption at the population level may be small [16,17,28]. In the present study, we examined the within-person change in bottled water and SSDB consumption in Philadelphia, relative to nearby comparison cities, one year after Philadelphia’s SSDB tax. Our study adds to the small, but growing, literature on the longer-term impact of beverage taxes on consumption, utilizing a quasi-experimental difference-in-differences design in a population-based sample. 

## 2. Materials and Methods

### 2.1. Study Sample 

A random-digit-dialing phone survey was conducted December 2016–February 2017 in residents aged 18–64 who lived in Philadelphia, Pennsylvania (“Philly group”) and nearby comparison cities (Trenton, New Jersey; Camden, New Jersey; and Wilmington, Delaware, “non-Philly group”) [24]. A landline and cell phone dual-frame design was used [29]. The study was approved by Drexel University IRB under expedited review, and verbal informed consent of subjects was obtained by GfK, a professional survey firm contracted for data collection. Participants who responded to the baseline survey (N = 2767, 1514 in the Philly group and 1253 in the non-Philly group) were re-contacted approximately one year later for a follow-up survey (December 2017–February 2018). A total of 863 participants (N = 479 in the Philly group and N = 384 in the non-Philly group) responded to the follow-up survey (33% retention). Losses were due to no response after at least 7 call attempts (50%), refusal (total 46%, which was comprised of hard refusal, 28%, and requests to be added to do not call list, 18%), or other reasons (3% number not in service, illness, etc.). Participants were paid $20 at follow-up but were not paid at baseline.

Missing baseline socio-demographic characteristics were imputed using data collected during follow-up. If still missing, race, education and income per capita were imputed using census ZIP code data (<5% of participants) or stratified sample means (by age group, sex and race, <0.5% of participants). Lastly, 19 participants were excluded due to missing bottled water or SSDB consumption data during baseline or follow-up, and two participants were excluded due to unreasonably extreme consumption of SSDB (i.e., more than 10,000 ounces during the past 30 days).

In order to evaluate changes in bottled water and SSDB consumption before and after the tax, participants were excluded if their baseline survey occurred after 15 January 2017 (mid-month after the tax took effect 1 January 2017). We did not exclude the entire month of January 2017 to maintain the sample size. The final analytic sample included 515 participants, which represented 60% of the follow-up sample (357 in Philly and 158 in non-Philly). Details on the number of participants by site and time period are shown in Table S1 and Figure S1. The analytic sample was similar to those who lost to follow-up (Table S2) and roughly comparable to the Philly census characteristics [30].

In post-hoc power analysis, we determined that our analytic sample size was powered to detect a quite small difference-in-difference (Philly compared to non-Philly, pretax vs. post-tax) in SSB beverage consumption: approximately three times per week (or 12.1 times/month or 209 ounces/month), fixing power at 0.8 and two-sided alpha 0.05 (Figure S2). This absolute detectable effect size was similar to the effects observed in Berkeley California (−16.5 times/month [27]) and in another study in Philadelphia (−10 to −11 times/month) [23].

We characterize a detectable effect of three times per week as ‘quite small’, based on the public health significance of an absolute change in consumption. Three times per week is less than one-half of a commonly used SSB threshold that has been linked to health conditions [31,32,33]: SSB consumption >= seven times per week (one time per day or >=30 times per month). While our sample has sufficient power for a reasonable change on an absolute scale, our sample does not have sufficient power for a reasonable change on a relative scale. Given that the magnitude of relative change depends on baseline prevalence and we observed low baseline consumption (approximately 5.56 times per week), a small absolute change of three times per week would translate to an extremely high relative change: 53% (observed Philadelphia baseline SSB consumption was approximately 5.56 times per week—[detectable absolute change three times per week]/5.56 = 0.53). A relative change of that magnitude would be unreasonable to expect. If a more reasonable relative change of 20% is expected, it would require a difference of 1.1 SSB per week, which we do not have the power to detect. See other work for discussion of relative or absolute changes [34].

### 2.2. Measures

The survey collected age, sex, race, education, income, height, weight, health status, smoking, and alcohol use. Missing baseline characteristics were imputed using follow-up data, census data (<5% of participants) and sample mean by age group, sex and race (<0.5% of participants). Self-reported bottled water and SSDB consumption (i.e., regular soda, regular fruit beverage, regular energy beverage, diet soda, diet fruit beverage, and diet energy beverage) were measured using a modified version of the validated 15-item beverage intake questionnaire (BEVQ-15) (test-retest correlation R^2^ = 0.98, *p* ≤ 0.01) [35]. Detailed survey questions and beverage size assumptions were described previously [24]. The outcomes included changes in 30-day consumption frequencies (continuous), changes in 30-day consumption ounces (continuous), daily consumption (yes/no), defined as more than 30 times in the past 30 days, and three discrete categories of direction of overall change (no change, increase, decrease).

### 2.3. Statistical Analysis 

Baseline sociodemographic characteristics and unadjusted outcomes were summarized for Philly and non-Philly. In a difference-in-differences analysis, the change in bottled water and SSDB consumption in Philly from baseline to the 12-month follow-up estimated relative to that of the comparison cities (details about the difference-indifferences regressions are available in Table S3). The distribution of changes in 30-day consumption frequencies or ounces was relatively normal and linear regression was performed. Logistic regression was performed to assess the change in the odds of daily consumption, and multinomial logistic regression was conducted to assess the direction of overall change (no change, increase, decrease). As an alternative approach, in sensitivity analyses we also tested in separate log-binomial models to estimate the relative risks [36].

All analyses were adjusted for baseline age, sex, race, education, income, body mass index (BMI), health status (chronic conditions and general health status), smoking, alcohol use, and whether the resident lived in a ZIP code that is on the Philly border (to control for potential cross-border shopping). We also adjusted for week/month of the baseline survey to account for seasonality.

## 3. Results

Participants’ baseline characteristics are described in Table 1. The final analytic sample included 515 participants (357 in Philly and 158 in non-Philly). Forty-nine percent were male, 52.2% were white, 42.5% were older than 50 years, 19.8% had income per capita lower than $15,000, 28.0% had educational attainment at high school level or lower, 28.7% were obese, 13.8% reported fair or poor self-rated health status, 40.4% reported having been told by a health professional that they had chronic health condition, 15.5% were currently smoking and 23.7% had higher alcohol use. About two-thirds of the participants were reached by cell phone and one-third lived near the Philly border at baseline. Table 1 shows that Philly and non-Philly participants mostly shared demographic and health characteristics. Philly was less advantaged (according to socioeconomic status and health variables) but, across most variables, the differences were small.

Bottled water and SSDB consumption during baseline and follow-up are summarized in Table 2 and one-year within-person changes are summarized in Table 3. Due to wide distribution of the consumption data, the SDs for the within-person changes in consumption frequency and ounces are extremely large. The bivariate Spearman correlation between the baseline and one-year consumption of SSBs was approximately 0.60 (not shown in tables). We observed declines in SSB consumption in Philly and non-Philly. The Philly group had a relatively higher percentage decrease and lower percentage increase in SSB consumption compared with the non-Philly group. Consumption of diet beverages remained almost the same for both groups. One year after the tax, in Philly and non-Philly, similar proportions of participants had an increase or a decrease in bottled water consumption. The majority of participants in Philly and non-Philly had no change in the category of being a daily drinker of bottled water or SSDB. Very few participants changed their daily consumption behavior, and the results for this outcome were unstable (Tables S4 and S5). 

The adjusted difference-in-differences analysis results are shown in Table 4. No significant changes were found in consumption over time between Philly and non-Philly. For example, the estimated difference-in-difference in 30-day SSB consumption frequency and ounces in this study was small (−3.03 times per month or −51.65 ounces) and confidence intervals were very wide and *p*-values were approximately 0.5 (Table 4-A). A small sample size and high variability led to high uncertainty in within-person adjusted results (all *p*-values ≥ 0.11). Results were insensitive to models without adjustment for covariates (difference-in-differences results without adjustment for covariates were largely the same) and results were insensitive to using log-binomial models (multinomial results were similar and inference was unchanged, Table S6).

Sensitivity to different definitions of the pre-tax period are presented in Figure S3; the point estimates were unstable and results were not statistically significant. Furthermore, we tested sensitivity to treatment contamination and/or lower-dose tax exposure in a few ways. First, we examined sensitivity of results to excluding participants who lived on the Philly border (43.4% of the Philly sample and 6.3% of the non-Philly sample resided in a ZIP code that touched the Philly city border, excluded N = 165); results remained null (Figure S3). Next, we excluded Philly residents who regularly consumed SSB and reported only purchasing those beverages outside of Philly at follow-up (excluded N = 65); results also remained null (Figure S3).

## 4. Discussion

We conducted a population-based longitudinal study using a random-digit-dialing phone survey of 515 participants in Philly and outside of Philly to estimate the impact of the Philadelphia beverage tax on consumption one year after implementation. In Philly and outside of Philly, there was a very small decline in monthly SSB consumption (for example, −3.0 times or −50 ounces). In Philly, a slightly higher proportion of participants decreased their monthly SSB consumption (39.2% in Philly vs. 33.5% non-Philly) and a slightly lower proportion increased their monthly SSB consumption (38.9% in Philly vs. 43.0% non-Philly). However, the majority of the participants (around 80% in both groups) did not change daily consumption of SSBs. After covariates adjustment, there were no differences between Philly and non-Philly in within-person change in consumption regardless of the type of beverage or operationalization (daily, continuous ounces, continuous frequency of consumption). Finally, the small effect sizes found in this study suggest that the tax may not have a substantive impact on population level health. We were unable to test whether the tax’s effect differed in sub-populations.

Notable strengths of our study were that we assessed changes in residents’ consumption, utilized a cohort and contrasted within-person changes in the tax site vs. multiple comparison areas in neighboring states, and that our study was population-based and thus designed to assess the impact of the tax on the general population. Compared to the census, our analytic sample was slightly older and had higher median income, nevertheless it was roughly comparable on other census characteristics [30]. Furthermore, the prevalence of SSB consumption in our analytic sample (e.g., 24% daily SSB consumption and 11% daily soda consumption) roughly aligned with the prevalence of SSBs reported in other population-based samples within Philadelphia [38]. 

To date, only one Philadelphia tax study has evaluated the tax’s impact on consumption. Cawley et al. enrolled shoppers via exit interviews and collected beverage consumption data before and one year after the tax [23]. They over-sampled disadvantaged residents (sample was over 60% African-American, majority low income and eligible for food assistance programs, most households had children). Their analytic sample of approximately 400 participants had high consumption of soda (approximately one-half of the sample consumed soda daily). One year after the tax, there was a small decrease in adult soda consumption in Philadelphia (−7.2 times per month) whereas consumption increased at comparison sites (+10.3 times per month). This resulted in a −10 to −11 times per month adjusted difference-in-difference (DID) decline in adult soda consumption frequency, but not for other beverages [23]. To summarize, differences in recent results from Cawley et al. and our study were likely due to Cawley et al. utilizing a population sub-sample (majority disadvantaged, adults living with children, and adults with a high consumption of SSB at baseline). 

Experience in Berkeley also suggested that the beverage tax has little effect on the general population, but does have an impact within disadvantaged subgroups and among persons with high SSB consumption. In Berkeley, a population-based study by Silver et al. (2017) conducted one year following the tax (which was also limited by small sample sizes, large standard errors and low baseline consumption) found very small non-significant declines in taxed beverage consumption (compared to the non-Berkeley area, −25 ounces per month or −0.85 ounces per day, consumption frequency was not reported in this study) [17]. However, another Berkeley study conducted by Lee et al. (2019) three years after the tax enrolled a more demographically diverse sample of participants who drank more SSBs. They found SSB declined in Berkeley but not at the comparison site, resulting in modest difference-in-difference (DID) reductions in SSB consumption (declined −0.55 times per day, roughly −16.5 times per month) and increased consumption of water (+1.02 times per day, roughly 30.6 times per month) [27]. 

Our current study suggests that the tax’s impact on general population consumption of SSB may have been more modest than was originally anticipated. Early prediction/simulation studies, supported by theories of price-elasticity, forecasted a fairly large impact of the beverage tax on SSB consumption [12]. However, most of the simulations did not account for many negative pressures on health decision making, such as persistent consumer preferences, intensification of marketing by SSB manufacturers or retailers, or availability of cross-border shopping. Thus, early work may have over-estimated the simulated impact of the tax [39,40]. Furthermore, recent studies have reported large and significant reductions in sales of/demand for taxed beverages in Philadelphia vs. comparison areas. [16,22,23]. Results from consumption studies like ours may be discordant with sales/demand studies for a number of reasons. Sales data do not capture out-of-home consumption (e.g., restaurants, vending machines, friends’ homes) and typically represent only larger-format retail establishments. Additionally, sales data studies have difficulty accounting for cross-border purchases; consumers may increase purchases of taxed beverages in nearby jurisdictions where beverages are not taxed [16]. Indeed, the two studies that examined Philadelphians’ sales/demand for taxed beverages estimated that the impact of the tax on SSB demand was attenuated due to cross-border shopping [16,22]. The exit interview conducted by Cawley et al. found that Philadelphians were not more likely to travel outside of the city to shop due to the tax, but when they did shop outside, they purchased more taxed beverages [23]. Because our analyses focused on consumption, the results will not be biased by cross-border shopping. Nevertheless, for descriptive purposes, we collected the taxed beverage shopping location (i.e., only in Philly, only outside of Philly, or both) among a subset of the sample; and those results also suggest increases in cross-border shopping. Among the subsample of Philadelphians asked the question, the proportion who only purchased SSDB outside Philly increased from 15.1% (37/245) at baseline to 25.6% (65/254) after one year, and shopping in and outside of Philly increased slightly from 25.7% (63/245) to 32% (82/254). 

This study has several limitations. First, our sample size was relatively small, and due to the large variance in consumption data, this study was only powered to detect large changes relative to the baseline consumption. The Cawley study had a smaller sample than ours, but had sufficient power to detect statistically significant changes due to observing a larger average effect (DID −17.5 SSB per month vs. our very small DID −3.03 SSB per month) and due to enrolling a relatively homogeneous sample. Studies conducted in population-based samples reported similar variability in beverage consumption as observed in our study [25,27]. Interestingly, Cawley et al. (2019) conducted a similar exit interview in Oakland, California in a small sample with similarly low baseline beverage consumption (22.63 times per month) as in our study, and they reported no substantial changes in overall consumption of SSB for either adults or children one year after the tax [28]. Second, our results may be generalizable to other large US cities with demographically diverse populations, but are not likely generalizable to subpopulations, such as high consumers of soda and lower income residents. Third, attrition in the follow-up survey was higher among Hispanic, lower income, lower education participants who may be more likely to consume more SSB and be price-sensitive [41]. This pattern was similar for Philly and non-Philly, except that a larger share of non-Whites were loss to follow-up in non-Philly. Hispanic and Black participants had higher SSB consumption at baseline and follow-up (data not shown). If participants with high SSB consumption would react to the tax more than others, the impact of the tax might be overestimated. Fourth, with only one pre-tax time period, we were unable to test the parallel trends assumption of the difference-in-differences method (which assumes the average outcomes for treated and control groups would have followed parallel paths over time). Fifth, we adjusted for differences in baseline characteristics in the Philly group and the non-Philly group by including covariates in the difference-in-differences model and time invariant characteristics were controlled for via within-person change analyses. Nevertheless, unobserved time-varying confounding remains a possibility. Finally, self-reported consumption of beverages likely has measurement error. We adapted a validated beverage questionnaire and results represent within-person changes in consumption, thus accounting for correlated errors within an individual’s self-report.

## 5. Conclusions

Limitations notwithstanding, this study is among the first to suggest that the impact of the tax on consumption may have been more modest than anticipated. Reasons for a modest impact may be the continued low price of SSB relative to healthier beverages (such as milk and 100% vegetable juice), high availability, aggressive marketing [9,10,11,42], as well as tax avoidance (purchasing SSB from retailers within Philadelphia who chose to not raise the price of SSB or purchasing from retailers outside of Philadelphia). Taken in context with other studies, it is important to conduct further research to understand the full effects of the tax on consumption, including research in larger population-based samples across multiple cities, as well as, among high-risk sub-populations. The Philadelphia beverage tax proposal was introduced with the explicit goal of generating revenue by taxing a luxury/non-essential item that could be used to finance universal pre-kindergarten programs and improvements to parks and recreation facilities [15]. Future studies could also examine whether health benefits are detectable from these health-promoting investments. 

## Figures and Tables

**Table 1:** Baseline characteristics of participants who responded to the baseline survey before 15 January 2017 and the one-year follow-up survey ^1^, N = 515.

Baseline Characteristics	Total	Philly	Non-Philly
	N = 515	N = 357	N = 158
Age, years, n (%)			
18 to 20	30 (5.8)	21 (5.9)	9 (5.7)
21 to 34	124 (24.1)	87 (24.4)	37 (23.4)
35 to 49	142 (27.6)	99 (27.7)	43 (27.2)
50 to 64	219 (42.5)	150 (42.0)	69 (43.7)
Male, n (%)	253 (49.1)	167 (46.8)	86 (54.4)
Race/ethnicity, n (%)			
Hispanic	31 (6.0)	21 (5.9)	10 (6.3)
Black	186 (36.1)	149 (41.7)	37 (23.4)
White	269 (52.2)	168 (47.1)	101 (63.9)
Other	29 (5.6)	19 (5.3)	10 (6.3)
Household income per capita, n (%)			
>$50,000	98 (19.0)	60 (16.8)	38 (24.1)
$30,001–$50,000	152 (29.5)	105 (29.4)	47 (29.7)
$15,001–$30,000	163 (31.7)	112 (31.4)	51 (32.3)
≤$15,000	102 (19.8)	80 (22.4)	22 (13.9)
Household income per capita, mean (STD)	33700 (23900)	32500 (23800)	36700 (23900)
Highest education, n (%)			
High school/GED	144 (28.0)	105 (29.4)	39 (24.7)
Technical school/2 year college	126 (24.5)	89 (24.9)	37 (23.4)
4 year college	126 (24.5)	85 (23.8)	41 (25.9)
Graduate school	119 (23.1)	78 (21.8)	41 (25.9)
BMI category, n (%)			
<25 (underweight ^2^ or normal)	205 (39.8)	144 (40.3)	61 (38.6)
≥25–<30 (overweight)	162 (31.5)	106 (29.7)	56 (35.4)
≥30 (obese)	148 (28.7)	107 (30.0)	41 (25.9)
BMI, mean (STD)	28.0 (7.2)	28.0 (7.0)	27.9 (7.5)
Self-rated health, n (%)			
Poor or fair	71 (13.8)	53 (14.8)	18 (11.4)
Good, very good, excellent	444 (86.2)	304 (85.2)	140 (88.6)
Chronic conditions, n (%) ^3^	208 (40.4)	137 (38.4)	71 (44.9)
Currently smoking, n (%)	80 (15.5)	53 (14.8)	27 (17.1)
Alcohol use ^4^, n (%)			
Higher	122 (23.7)	84 (23.5)	38 (24.1)
Lower	206 (40.0)	136 (38.1)	70 (44.3)
None	157 (30.5)	116 (32.5)	41 (25.9)
Younger than 21 years old ^4^	30 (5.8)	21 (5.9)	9 (5.7)
Reached by cell phone, n (%)	341 (66.2)	224 (62.7)	117 (74.1)
Living near Philly border at baseline ^5^, n (%)	165 (32.0)	155 (43.4)	10 (6.3)
SSB or diet purchase location at baseline			
Participants who were asked	296 (57.5)	245 (68.6)	51 (32.3)
Purchased only in Philly	146 (28.3)	145 (40.6)	1 (0.6)
Purchased only outside of Philly	76 (14.8)	37 (10.4)	39 (24.7)
Purchased in and outside of Philly	74 (14.4)	63 (17.6)	11 (7.0)
Participants who were not asked ^6^	219 (42.5)	112 (31.4)	107 (67.7)
SSB or diet purchase location at follow-up			
Participants who were asked	324 (62.9)	254 (71.1)	70 (44.3)
Purchased only in Philly	107 (20.8)	107 (30.0)	0 (0.0)
Purchased only outside of Philly	108 (21.0)	65 (18.2)	43 (27.2)
Purchased in and outside of Philly	109 (21.2)	82 (23.0)	27 (17.1)
Participants who were not asked ^6^	191 (37.1)	103 (28.9)	88 (55.7)
Week of baseline survey, n (%)			
1	164 (31.8)	117 (32.8)	47 (29.7)
2	60 (11.7)	33 (9.2)	27 (17.1)
3	49 (9.5)	31 (8.7)	18 (11.4)
4	84 (16.3)	56 (15.7)	28 (17.7)
5	109 (21.2)	78 (21.8)	31 (19.6)
6	49 (9.5)	42 (11.8)	7 (4.4)
Month of survey, n (%)			
Baseline Dec, follow-up Dec	183 (35.5)	121 (33.9)	62 (39.2)
Baseline Dec, follow-up Jan	149 (28.9)	98 (27.5)	51 (32.3)
Baseline Jan, follow-up Jan	183 (35.5)	138 (38.7)	45 (28.5)

^1^ Missing baseline characteristics were imputed using follow-up data, census data (<5% of participants) and sample mean by age group, sex and race (<0.5% of participants). ^2^ There were very few underweight (<2%), thus, they were grouped with normal weight. ^3^ Presence of a chronic condition was assessed by asking whether the participant was ever told by a doctor, nurse, or other health professional that they had at least one of the following: high blood pressure, high cholesterol, diabetes, or history of heart disease. ^4^ Alcohol use only asked if participants were aged >=21. Alcohol “higher” was defined as more than seven drinks for female or 14 drinks for male [37]. ^5^ Lived in ZIP code that is on the Philadelphia border. ^6^ Only asked among those who spent ≥1 day in Philly during the past 30 days and consumed regular or diet beverages more than three times per month. Abbreviation: GED, General Education Development test.

**Table 2:** Frequency and mean of bottled water, sugar-sweetened and diet beverage consumption during baseline and follow-up, N = 515.

Beverage		Philly (Tax) N = 357		*p*-Value ^1^	Non-Philly (no Tax) N = 158		*p*-Value ^1^
		Baseline	Follow-Up		Baseline	Follow-Up	
	**A. Total 30-day consumption frequency**						
**Mean (STD)**							
Bottled water		39.7 (107.7)	45.8 (124.7)	0.476	30.9 (53.8)	50.9 (198.1)	0.186
Sugar sweetened beverage ^2^		22.6 (40.5)	19.8 (32.2)	0.228	17.8 (34.7)	14.9 (24.4)	0.251
Regular soda		7.8 (18.1)	7.7 (15.6)	0.934	9.2 (20.9)	7.6 (16.3)	0.325
Regular fruit beverage		10.7 (27.3)	9.4 (20.9)	0.423	6.3 (22.1)	4.4 (9.9)	0.265
Regular energy beverage		4.1 (15.6)	2.7 (10.0)	0.108	2.3 (8.0)	3.0 (8.0)	0.223
Diet beverage ^2^		8.5 (21.5)	10.1 (26.6)	0.291	12.6 (35.5)	13.0 (35.8)	0.862
Diet soda		4.8 (14.9)	4.4 (13.1)	0.619	8.5 (24.4)	8.6 (29.2)	0.964
Diet fruit beverage		2.3 (10.4)	3.8 (19.8)	0.161	2.3 (9.4)	2.5 (12.3)	0.840
Diet energy beverage		1.5 (6.2)	1.8 (9.4)	0.540	1.8 (12.3)	1.9 (9.5)	0.866
**Median (range 25th-75th)**							
Bottled water		10.0 (0.0, 60.0)	20.0 (0.0, 60.0)		5.0 (0.0, 30.0)	8.0 (0.0, 60.0)	
SSB		6.0 (0.0, 28.0)	8.0 (0.0, 24.0)		4.0 (0.0, 20.0)	6.0 (1.0, 20.0)	
Diet beverage		0.0 (0.0, 4.0)	0.0 (0.0, 4.0)		0.0 (0.0, 6.0)	0.0 (0.0, 8.0)	
	**B. Total 30-day consumption in ounces**						
**Mean (STD)**							
Bottled water		793.8 (2153.4)	915.2 (2494.5)	0.476	618.7 (1076.5)	1018.1 (3961.1)	0.186
Sugar sweetened beverage ^2^		357.3 (753.3)	310.8 (533.3)	0.264	284.6 (627.5)	232.1 (459.1)	0.230
Regular soda		115.8 (298.9)	130.3 (308.0)	0.438	150.6 (349.8)	122.4 (346.3)	0.313
Regular fruit beverage		176.5 (561.0)	135.6 (297.6)	0.181	94.3 (406.9)	61.5 (139.7)	0.309
Regular energy beverage		65.0 (226.6)	44.9 (153.8)	0.133	39.7 (169.4)	48.2 (121.4)	0.470
Diet beverage ^2^		120.8 (308.6)	141.6 (347.4)	0.256	217.3 (678.6)	197.4 (508.9)	0.679
Diet soda		61.7 (189.8)	63.6 (181.1)	0.835	142.3 (536.5)	124.5 (383.9)	0.681
Diet fruit beverage		31.6 (142.4)	47.3 (230.0)	0.199	42.0 (199.1)	35.1 (192.4)	0.686
Diet energy beverage		27.4 (132.3)	30.7 (140.5)	0.708	33.0 (245.7)	37.7 (195.0)	0.798
**Median (range 25th-75th)**							
Bottled water		200.0 (0.0, 1200.0)	400.0 (0.0, 1200.0)		100.0 (0.0, 600.0)	160.0 (0.0, 1200.0)	
SSB		80.0 (0.0, 336.0)	96.0 (0.0, 340.0)		70.0 (0.0, 300.0)	80.0 (20.0, 264.0)	
Diet beverage		0.0 (0.0, 60.0)	0.0 (0.0, 80.0)		0.0 (0.0, 90.0)	0.0 (0.0, 120.0)	

^1^*p*-values were estimated from paired *t*-tests. ^2^ “Soda” refers to soft drinks, “fruit” refers to fruit drinks that are not 100% fruit juice, and “energy” refers to energy or sports drinks. Due to small N, beverage subgroups are not presented in the median tables.

**Table 3:** Within-person change in bottled water, sugar-sweetened and diet beverage consumption (unadjusted for covariates), N = 515.

Type of Beverage		Philly (Tax)	Non-Philly (No Tax)	Philly (Tax)	Non-Philly (No Tax)
		N = 357	N = 158	N = 357	N = 158
		**A-i. Within-person changes in 30-day consumption frequency**		**B-i. Within person changes in 30-day consumption ounces**	
Bottled water	Mean (STD)	6.1 (160.6)	20.0 (188.8)	121.3 (3211.8)	399.4 (3775.1)
SSB	Mean (STD)	−2.8 (43.9)	−2.9 (31.5)	−46.5 (785.9)	−52.5 (547.8)
Diet beverage	Mean (STD)	1.5 (26.9)	0.5 (33.5)	20.9 (346.6)	−19.9 (603.1)
					
Bottled water	Median (25th-75th)	0.0 (−12.0, 27.0)	0.0 (−10.0, 12.0)	0.0 (−240.0, 540.0)	0.0 (−200.0, 240.0)
SSB	Median (25th-75th)	0.0 (−7.0, 5.0)	0.0 (−4.0, 4.0)	0.0 (−72.0, 80.0)	0.0 (−50.0, 80.0)
Diet beverage	Median (25th-75th)	0.0 (0.0, 0.0)	0.0 (0.0, 1.0)	0.0 (0.0, 0.0)	0.0 (0.0, 20.0)
		**A-ii. Within-person frequency changed**		**B-ii. Within-person ounces changed**	
Bottled water	Decrease	119 (33.3%)	58 (36.7%)	119 (33.3%)	58 (36.7%)
	No change	93 (26.1%)	38 (24.1%)	93 (26.1%)	38 (24.1%)
	Increase	145 (40.6%)	62 (39.2%)	145 (40.6%)	62 (39.2%)
					
SSB	Decrease	140 (39.2%)	53 (33.5%)	140 (39.2%)	53 (33.5%)
	No change	78 (21.8%)	37 (23.4%)	76 (21.3%)	34 (21.5%)
	Increase	139 (38.9%)	68 (43.0%)	141 (39.5%)	71 (44.9%)
					
Diet beverage	Decrease	74 (20.7%)	30 (19.0%)	74 (20.7%)	32 (20.3%)
	No change	201 (56.3%)	88 (55.7%)	198 (55.5%)	84 (53.2%)
	Increase	82 (23.0%)	40 (25.3%)	85 (23.8%)	42 (26.6%)

**Table 4:** Difference-in-differences analysis (adjusted for covariates ^1^), N = 515.

**A. Change in Beverage Consumption Volume or Frequency in 12 Months after the Tax in Philadelphia Relative to that of the Comparison Cities (Philly vs. Non-Philly)**						
	**Mean Change in Frequency (Continuous)**	**95% CI**	***p*-Value**	**Mean Change in Ounces (Continuous)**	**95% CI**	***p*-Value**
Bottled water	−15.79	−51.37 to 19.79	0.384	−315.81	−1027.38 to 395.76	0.384
SSB	−3.03	−11.43 to 5.38	0.479	−51.65	−202.32 to 99.02	0.501
Diet beverage	0.40	−5.69 to 6.50	0.897	21.62	−70.94 to 114.17	0.646
**B. Odds Ratio of being in the Specified Category vs. no Change 12 Months after the Tax in Philadelphia Relative to that of the Comparison Cities (Philly vs. Non-Philly)**						
	**Change in Frequency (Increase/Decrease)**	**95% CI**	***p*-Value**	**Change in Ounces (Increase/Decrease)**	**95% CI**	***p*-Value**
Bottled water						
Decrease	0.77	0.44 to 1.35	0.365	0.77	0.44 to 1.35	0.365
Increase	0.92	0.53 to 1.59	0.769	0.92	0.53 to 1.59	0.769
SSB						
Decrease	1.15	0.62 to 2.12	0.653	1.01	0.54 to 1.87	0.987
Increase	0.74	0.41 to 1.33	0.315	0.62	0.34 to 1.12	0.113
Diet beverage						
Decrease	1.26	0.72 to 2.19	0.423	1.14	0.66 to 1.99	0.633
Increase	0.97	0.57 to 1.64	0.908	0.90	0.54 to 1.52	0.699

^1^ Results were adjusted for baseline age, sex, race, education, income, body mass index, health status, smoking, alcohol use, lived in ZIP code that is on the Philadelphia border (to control for potential cross-border shopping), and week of baseline survey (to control for seasonal trend).

## Supplementary Materials

The following are available online at https://www.mdpi.com/1660-4601/17/4/1336/s1, Figure S1: Sample selection flow chart; Figure S2: Power analysis for sugar-sweetened beverage consumption; Figure S3: Sensitivity analysis. Difference-in-differences analysis adjusted for covariates; Table S1: Number (%) of survey participants by time interval during follow-up survey; Table S2: Baseline characteristics of participants retained in the analytic sample and dropped out due to loss-to-follow-up; Table S3: Description of the difference-in-differences models. Table S4: Daily consumption of bottled water, sugar-sweetened and diet beverage during baseline and follow-up; Table S5: Within-person change in daily consumption of bottled water, sugar-sweetened and diet beverage during baseline and follow-up; Table S6: Difference-in-differences analysis (adjusted for covariates1): relative risk of being in the specified category 12 months after the tax in Philadelphia relative to that of the comparison cities.
