# Supplementary material for: Sugar-Sweetened and Diet Beverage Consumption in Philadelphia One Year after the Beverage Tax
Source: Int J Environ Res Public Health. 2020 Feb 19;17(4):1336. doi: 10.3390/ijerph17041336 (PMC7068482; doi:10.3390/ijerph17041336)
Supplement: Supplementary file 1 [file ijerph-17-01336-s001.pdf]

Title: Sugar-Sweetened and Diet Beverage Consumption in Philadelphia One Year after the Beverage Tax

Authors: Yichen Zhong (yz624@drexel.edu), Amy H. Auchincloss (aha27@drexel.edu), Brian K. Lee, Ryan M. McKenna, Brent A. Langellier

Author affiliation: Dornsife School of Public Health, Drexel University, Philadelphia, PA 19104

Supplementary Materials

List of tables and figures

Table S1. Number (%) of survey participants by time interval during follow-up survey.

Table S2. Baseline characteristics of participants retained in the analytic sample (N = 515) and dropped out due to loss-to-follow-up (N = 1904) .

Table S3. Description of the difference-in-differences models.

Table S4. Daily consumption of bottled water, sugar-sweetened and diet beverage during baseline and follow-up (N = 515).

Table S5. Within-person change in daily consumption of bottled water, sugar-sweetened and diet beverage during baseline and follow-up (N=515).

Table S6. Difference-in-differences analysis (adjusted for covariates): relative risk of being in the specified category 12 months after the tax in Philadelphia relative to that of the comparison cities (N = 515).

Figure S1. Sample selection flow chart.

Figure S2. Power analysis for sugar-sweetened beverage consumption.

Figure S3. Sensitivity analysis. Difference-in-differences analysis adjusted for covariates.

Table S1. Number (%) of survey participants by time interval[1] during follow-up survey.

	Philly	Non-Philly	Total
**1. All participants**			
Before Jan 1, 2017	229 (47.8%, 229/479)	120 (31.2%)	349 (40.4%)
Jan 1, 2017 to Jan 15, 2017	141 (29.4%)	45 (11.7%)	186 (21.5%)
Jan 16, 2017 to Jan 30, 2017	62 (12.9%)	149 (38.8%)	211 (24.4%)
After Jan 30, 2017	47 (9.8%)	70 (18.2%)	117 (13.6%)
Total	479	384	863
**2. Sample after excluding participants with missing data [2] or outliers [3]**			
Before Jan 1, 2017	219 (47.3%, 219/464)	113 (30.5%)	332 (39.8%)
Jan 1, 2017 to Jan 15, 2017	138 (29.7%)	45 (12.1%)	183 (21.9%)
Jan 16, 2017 to Jan 30, 2017	62 (13.4%)	146 (39.3%)	208 (24.9%)
After Jan 30, 2017	45 (9.7%)	67 (18.0%)	112 (13.4%)
Total	464	371	835
**3. Main analysis sample after excluding participants with baseline survey after Jan 15, 2018**			
Total	357	158	515

[1] Note that the time intervals are not equally spaced. Survey participants did not join the study at a constant rate and, thus, the time intervals were chosen in order to have sufficient numbers of participants in each time interval. [2] First, missing baseline characteristics were inputted using data collected during follow-up. Second, missing education and income per capita data were inputted using census data. Missing race were inputted by race consisted at least 50% of the census tract population. Missing per capita income were inputted by the per capita income in the census tract. Participants aged 18–20 at baseline were assigned high school/GED education if they lived in a census tract with more than 50% finished high school, or some high school if otherwise. Participants older than 20 at baseline were assigned college education if they lived in a census tract with more than 50% finished college, or high school/GED if otherwise. Finally, the rest of the missing baseline characteristics were inputted by sample means by age group, sex and race. Participants missing beverage consumption data during either baseline or follow-up were excluded. [3] Participants who reported consuming more than 10,000 ounces of sweetened or diet beverages during the past 30 days were identified as outliers and excluded from the main analysis. Only two participants were excluded due to unreasonably extreme values.

Table S2. Baseline characteristics of participants retained in the analytic sample (N = 515) and dropped out due to loss-to-follow-up (N = 1904) [1].

Baseline characteristics	Philly Retained	Philly Dropped-out	Non-Philly Retained	Non-Philly Dropped-out
	N = 357	N = 1035	N = 158	N = 869
Age, years, n (%)				
18 to 20	21 (5.9)	63 (6.1)	9 (5.7)	88 (10.1)
21 to 34	87 (24.3)	292 (28.2)	37 (23.4)	232 (26.7)
35 to 49	99 (27.7)	281 (27.1)	43 (27.2)	231 (26.6)
50 to 64	151 (42.2)	399 (38.6)	69 (43.7)	318 (36.6)
Male, n (%)	167 (46.6)	471 (45.5)	86 (54.4)	422 (48.6)
Race/ethnicity, n (%)				
Hispanic	21 (5.9)	169 (16.3)	10 (6.3)	134 (15.4)
Black	149 (41.7)	437 (42.2)	37 (23.4)	281 (32.3)
White	168 (47.1)	376 (36.3)	101 (63.9)	407 (46.8)
Other	19 (5.3)	53 (5.1)	10 (6.3)	47 (5.4)
Household income per capita, n (%)				
>$50,000	60 (16.8)	138 (13.3)	38 (24.1)	152 (17.5)
$30,001–$50,000	105 (29.4)	220 (21.3)	47 (29.7)	246 (28.3)
$15,001–$30,000	112 (31.4)	378 (36.5)	51 (32.3)	303 (34.9)
≤$15,000	80 (22.4)	299 (28.9)	22 (13.9)	168 (19.3)
Household income per capita, mean (STD)	32455.5 (23769.5)	28402.8 (22252.2)	36663.8 (23909.4)	32757.6 (23263.2)
Highest education, n (%)				
High school/GED	105 (29.4)	445 (43.0)	39 (24.7)	337 (38.8)
Technical school/2 year college	89 (24.9)	267 (25.8)	37 (23.4)	191 (22.0)
4 year college	85 (23.8)	185 (17.9)	41 (25.9)	215 (24.7)
Graduate school	78 (21.8)	138 (13.3)	41 (25.9)	126 (14.5)
BMI category, n (%)				
<25 (underweight[2] or normal)	144 (40.3)	328 (31.7)	61 (38.6)	319 (36.7)
≥25–<30 (overweight)	106 (29.7)	374 (36.1)	56 (35.4)	306 (35.2)
≥30 (obese)	107 (30.0)	333 (32.2)	41 (25.9)	244 (28.1)
BMI, mean (STD)	28.0 (7.0)	28.1 (6.5)	27.9 (7.5)	27.8 (6.3)
Self-rated health, n (%)				
Poor or fair	53 (14.8)	195 (18.8)	18 (11.4)	134 (15.4)
Good, very good, excellent	304 (85.2)	840 (81.2)	140 (88.6)	735 (84.6)
Chronic conditions, n (%)[3]	137 (38.4)	358 (34.6)	71 (44.9)	282 (32.5)
Currently smoking, n (%)	53 (14.8)	223 (21.5)	27 (17.1)	203 (23.4)
Alcohol use, n (%)				
Higher	84 (23.5)	211 (20.4)	38 (24.1)	177 (20.4)
Lower	136 (38.1)	322 (31.1)	70 (44.3)	313 (36.0)
None	116 (32.5)	439 (42.4)	41 (25.9)	291 (33.5)
Younger than 21 years old[4]	21 (5.9)	63 (6.1)	9 (5.7)	88 (10.1)
Reached by cell phone, n (%)	224 (62.7)	643 (62.1)	117 (74.1)	711 (81.8)
Living near Philly border at baseline[5], n (%)	155 (43.4)	451 (43.6)	10 (6.3)	39 (4.5)

Table S2. Footnote

[1] Missing baseline characteristics were inputted using follow-up data, census data (<5% of participants) and sample mean by age group, sex and race (<0.5% of participants). [2] There were very few underweight (<2%), thus, they were grouped with normal weight. [3] Presence of a chronic condition was assessed by asking whether the participant was ever told by a doctor, nurse, or other health professional that they had at least one of the following: high blood pressure, high cholesterol, diabetes, or history of heart disease. [4] Only participants aged >=21 were asked about alcohol consumption. Alcohol use "higher" was defined as more than seven drinks for female or 14 drinks for male.[37] [5] Lived in ZIP code that is on the Philadelphia border. Abbreviation: GED, General Education Development test.

Table S3. Description of the difference-in-differences models.

Linear regression for continuous outcomes
$\Delta Y = \beta_1 + \beta_2 Philly + \beta_j Cov_j$ ΔY is the within-person change in the volume or frequency of beverage consumption. $Philly$ is the treatment group ("Philly" vs. "non-Philly"). The coefficient of interest is β_2, which can be interpreted as the change in beverage consumption volume or frequency in 12 months after the tax in Philadelphia relative to that of the comparison cities.
Multinomial logistic regression for binary or categorical outcomes $\log\frac{\Pr(Y=\mathrm{m})}{\Pr(Y=No\ change)} = \beta_1 + \beta_2 Philly + \beta_j Cov_j$ Y is the categorical variable for within-person change beverage consumption behavior. $Philly$ is the treatment group ("Philly" vs. "non-Philly"). The coefficient of interest is $Exp(\beta_2)$, which can be interpreted as the difference in the odds of being in a specific category vs. no change 12 months after the tax in Philadelphia relative to that of the comparison cities.
Logistic regression for binary outcome $\log\frac{\Pr(\mathrm{Y_i}=1)}{\Pr(\mathrm{Y_i}=0)} = \beta_1 + \beta_2 \mathrm{Period_i} + \beta_3 \mathrm{Philly_i} + \beta_4 \mathrm{Period_i} \times \mathrm{Philly_i} + \beta_j \mathrm{Cov_j}$ Y is the variable for daily consumption (1or 0). $Period$ is the time during which the outcome is measured ("baseline" vs. "12-month follow-up"). $Philly$ is the treatment group ("Philly" vs. "non-Philly"). i denotes the individual participants. The coefficient of interest is $Exp(\beta_4)$, which can be interpreted as the change in the odds of daily consumption 12 months after the tax in Philadelphia relative to that of the comparison cities.

Table S4. Daily consumption of bottled water, sugar-sweetened and diet beverage during baseline and follow-up (N = 515).

	Philly (Tax) N = 357		Non-Philly (no Tax) N= 158	
**Beverage**	**Baseline**	**Follow-Up**	**Baseline**	**Follow-Up**
Bottled water	145 (40.6%)	158 (44.3%)	53 (33.5%)	56 (35.4%)
Sugar sweetened beverage [1]	89 (24.9%)	82 (23.0%)	35 (22.2%)	24 (15.2%)
Regular soda	39 (10.9%)	29 (8.1%)	20 (12.7%)	11 (7.0%)
Regular fruit beverage	41 (11.5%)	43 (12.0%)	11 (7.0%)	8 (5.1%)
Regular energy beverage	18 (5.0%)	11 (3.1%)	3 (1.9%)	7 (4.4%)
Diet beverage [1]	34 (9.5%)	45 (12.6%)	23 (14.6%)	21 (13.3%)
Diet soda	21 (5.9%)	21 (5.9%)	16 (10.1%)	15 (9.5%)
Diet fruit beverage	14 (3.9%)	14 (3.9%)	6 (3.8%)	6 (3.8%)
Diet energy beverage	6 (1.7%)	10 (2.8%)	2 (1.3%)	4 (2.5%)

[1] "Soda" refers to soft drinks, "fruit" refers to fruit drinks that are not 100% fruit juice, and "energy" refers to energy or sports drinks. Due to small N, beverage subgroups are not presented in the main tables.

Table S5. Within-person change in daily consumption of bottled water, sugar-sweetened and diet beverage during baseline and follow-up (N=515).

**Beverage**		**Philly (Tax)** N = 357	**Non-Philly (no Tax)** N = 158
Bottled water	No change	238 (66.7%)	111 (70.3%)
	Became daily drinker	66 (18.5%)	25 (15.8%)
	Became non-daily drinker	53 (14.8%)	22 (13.9%)
SSB	No change	278 (77.9%)	131 (82.9%)
	Became daily drinker	36 (10.1%)	8 (5.1%)
	Became non-daily drinker	43 (12.0%)	19 (12.0%)
Diet beverage	No change	318 (89.1%)	146 (92.4%)
	Became daily drinker	25 (7.0%)	5 (3.2%)
	Became non-daily drinker	14 (3.9%)	7 (4.4%)

Table S6. Difference-in-differences analysis (adjusted for covariates [1]): relative risk of being in the specified category 12 months after the tax in Philadelphia relative to that of the comparison cities (N = 515).

	**Change in Frequency (Increase/Decrease) (Philly vs. Non-Philly)**	**95% CI**	***p*-Value**	**Change in ounces (Increase/Decrease) (Philly vs. Non-Philly)**	**95% CI**	***p*-Value**
Bottled water						
Decrease	0.87	0.66 to 1.15	0.342	0.87	0.66 to 1.15	0.342
Increase	1.05	0.81 to 1.36	0.718	1.05	0.81 to 1.36	0.718
SSB						
Decrease	1.12	0.90 to 1.39	0.301	1.16	0.92 to 1.47	0.206
Increase	0.80	0.62 to 1.03	0.081	0.78	0.62 to 1.00	0.052
Diet beverage						
Decrease	1.27	0.84 to 1.91	0.256	1.21	0.81 to 1.81	0.352
Increase	0.99	0.69 to 1.41	0.960	0.92	0.65 to 1.31	0.650

[1] Results were adjusted for baseline age, sex, race, education, income, body mass index, health status, smoking, alcohol use, survey method (cellphone vs. landline), if they lived in ZIP code that is on the Philadelphia border (to control for potential cross-border shopping), and week of baseline survey (to control for seasonal trend).

Figure S1. Sample selection flow chart.

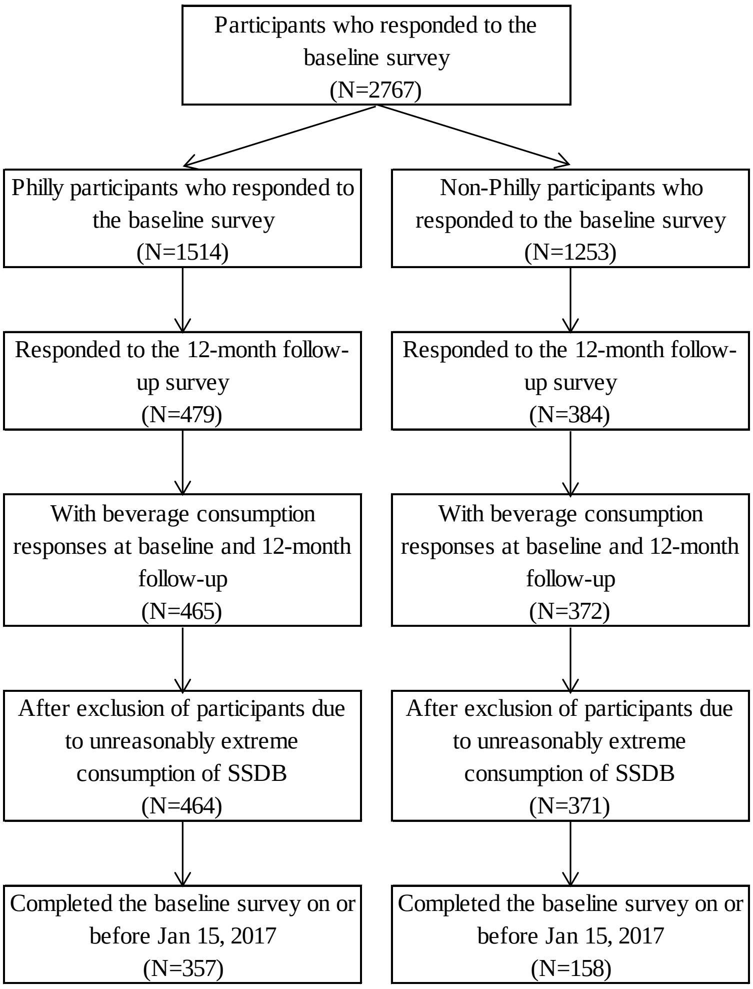

Figure S2. Power analysis for sugar-sweetened beverage consumption.

A. Detectable effect size vs. power in a two-sample t-test for a normally distributed outcome with a standard error of 45 (approximately the standard error for within-person change in monthly sugar-sweetened beverage consumption frequency in the Philly group) and sample size of 515 (357 in one group, 158 in another group).

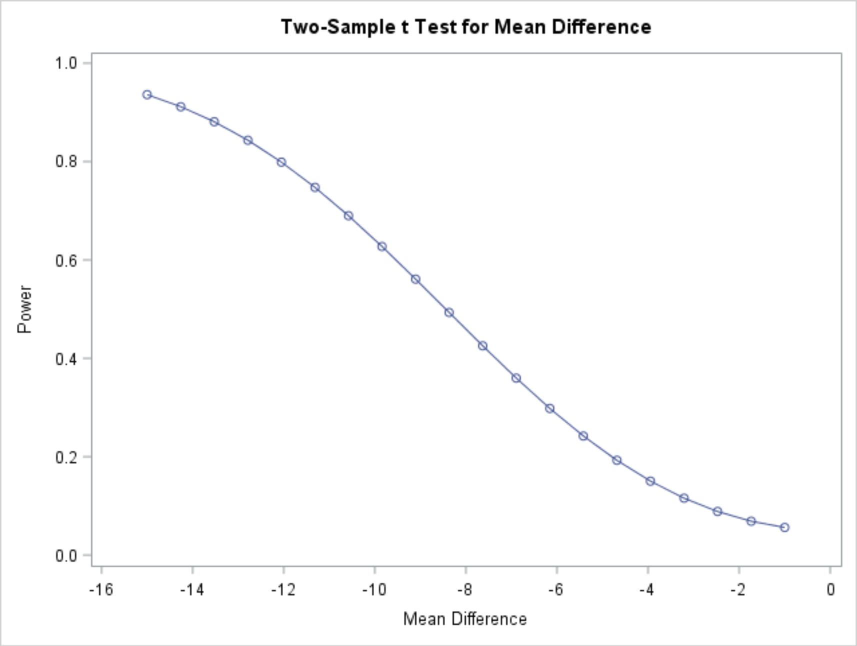

B. Detectable effect size vs. power in a two-sample t-test for a normally distributed outcome with a standard error of 780 (approximately the standard error for within-person change in monthly sugar-sweetened beverage consumption ounces in the Philly group) and sample size of 515 (357 in one group, 158 in another group).

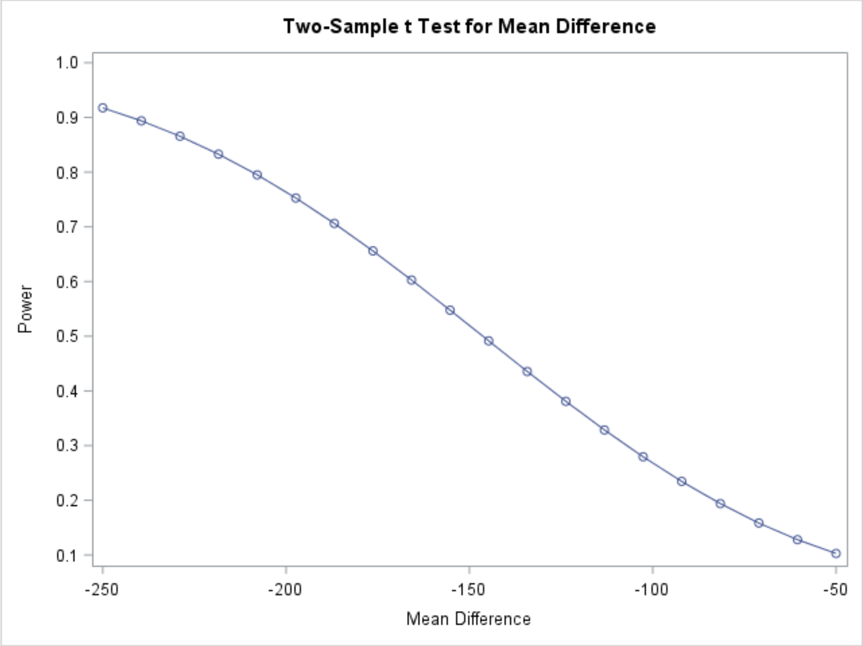

Figure S3. Sensitivity analysis. Difference-in-differences analysis adjusted for covariates.

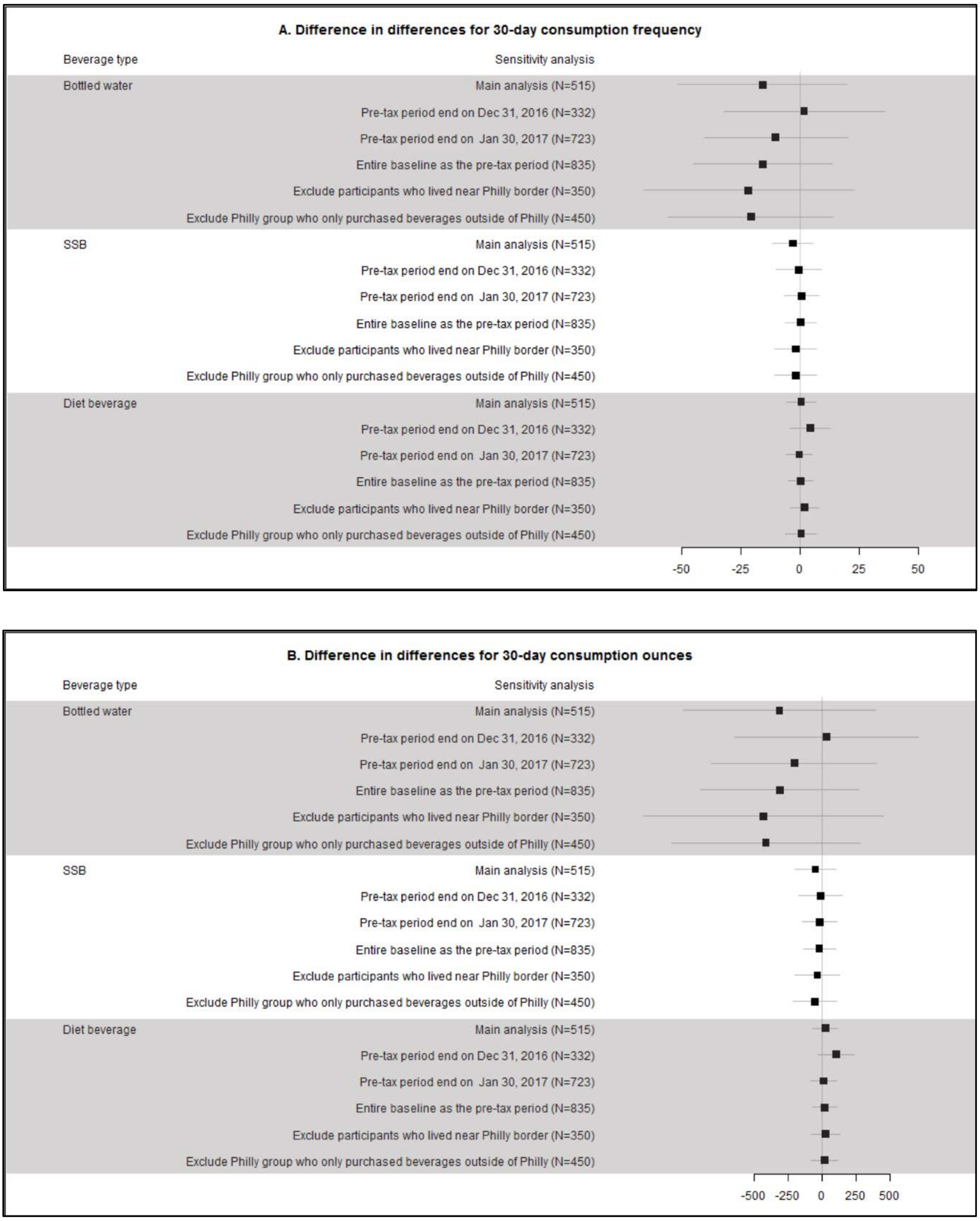

(Continuation of Figure S3. Sensitivity analysis. Difference-in-differences analysis adjusted for covariates.)

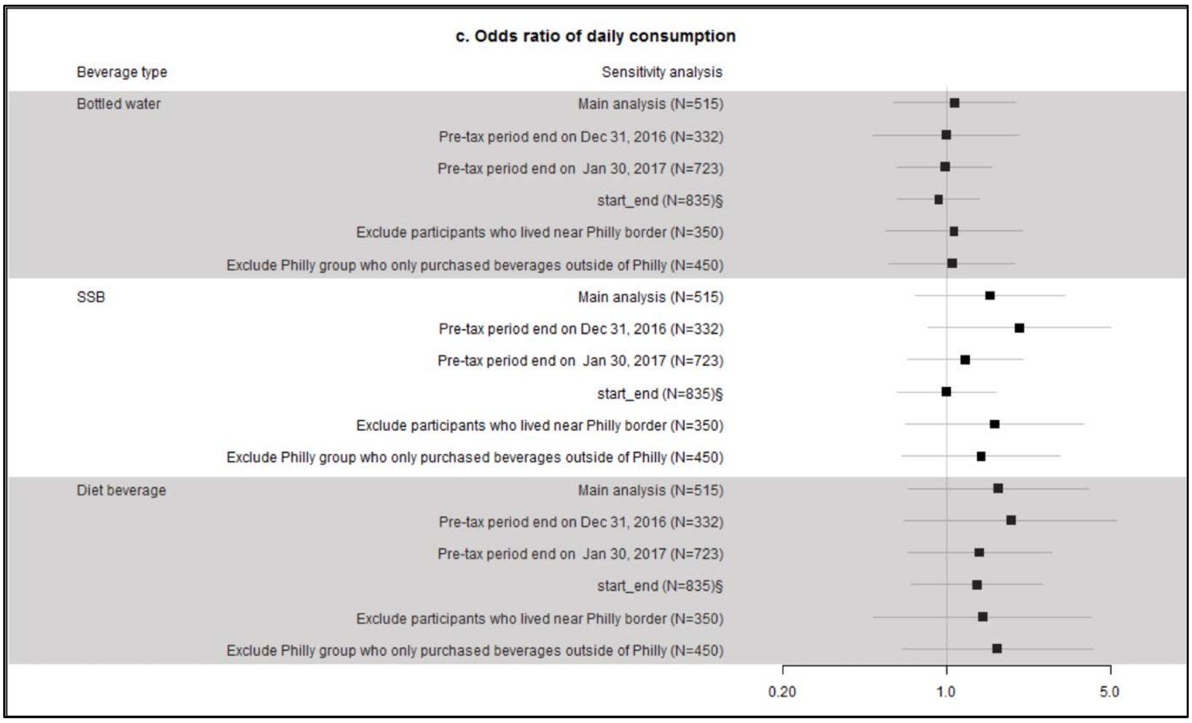

*Only a few participants changed the daily consumption behavior, the odds ratios of daily consumption are unstable.
